# Draft Genome Sequence of Bacillus marisflavi CK-NBRI-03, Isolated from Agricultural Soil

**DOI:** 10.1128/MRA.00044-20

**Published:** 2020-02-13

**Authors:** Mayank Gupta, Puneet Singh Chauhan, Sudhir K. Sopory, Sneh L. Singla-Pareek, Nidhi Adlakha, Ashwani Pareek, Charanpreet Kaur

**Affiliations:** a International Centre for Genetic Engineering and Biotechnology, New Delhi, India; b Microbial Technologies Division, CSIR-National Botanical Research Institute, Lucknow, Uttar Pradesh, India; c Regional Centre for Biotechnology, NCR Biotech Science Cluster, Faridabad, Haryana, India; d School of Life Sciences, Jawaharlal Nehru University, New Delhi, India; University of Rochester School of Medicine and Dentistry

## Abstract

Here, we report the 4.34-Mb draft genome assembly of Bacillus marisflavi CK-NBRI-03 (or P3), a Gram-positive bacterium, with an average G+C content of 48.66%. P3 was isolated from agricultural soil from the Badaun (midwestern plain zone) region of Uttar Pradesh, India.

## ANNOUNCEMENT

Bacillus marisflavi CK-NBRI-03 (P3) was isolated from a wheat field located in the Badaun region of Uttar Pradesh, India, while the microbial diversity of that area was explored post-wheat harvest. Preliminary 16S rRNA gene sequencing studies using the 27F (5′-AGAGTTTGATCMTGGCTCAG-3′) and 1492R (5′-TACGGYTACCTTGTTACGACTT-3′) primers (1) revealed 99.77% similarity (with 90% query coverage) of P3 to Bacillus marisflavi TF-11 (JCM 11544), a carotenoid-producing bacterium isolated from seawater (2). Although bacteria belonging to the Bacillus genus are well-known plant growth promoters, information regarding the involvement of any B. marisflavi strain in plant growth promotion is lacking. Therefore, sequencing of the P3 genome was undertaken to investigate its genomic features and to assess the potential role of this isolated *B. marisflavi* strain in influencing plant growth.

P3 was isolated from agricultural soil per a protocol described previously (3). For whole-genome sequencing studies, the strain was preserved as glycerol stock after initial isolation and purification and then restreaked onto a nutrient agar plate, and a single colony was inoculated in the nutrient broth. The culture was then grown at 28°C for 24 h, and harvested cells were subsequently used for genomic DNA isolation, carried out using the GenElute bacterial genomic DNA kit (Sigma-Aldrich). This was followed by library preparation using the NEBNext Ultra DNA library prep kit for Illumina (New England Biolabs, Ipswich, MA) as per the manufacturer’s instructions.

The Illumina HiSeq 2500 platform with 100-bp paired-end reads was used for the sequencing of the P3 genome, which generated a total of 949.46 Mb of raw reads. From these, Illumina adapter sequences were removed using Cutadapt version 1.14 (4). Low-quality (Q < 30) reads were filtered out using Sickle version 1.33 (5), and duplicate reads were removed using FastUniq 1.1 (6). After preprocessing, we obtained 857.40 Mb of clean paired-end reads at ∼200× genome coverage and with an average DNA G+C content of 48.66%. The reads were separately assembled using Velvet version 1.2.10 (7) and MaSuRCA version 2.2.1 (8) tools. The resulting assemblies were subsequently merged using GAA version 1.0 (9). Thereafter, PAGIT version 1 (10) was used for the scaffolding of the merged assembly. Sixteen scaffolds containing a total of 4,344,737 bp with an *N*_50_ value of 696,266 bp were obtained. The average scaffold length was 271,546 bp, and the longest and shortest scaffolds were 1,820,907 bp and 1,001 bp, respectively.

The final draft genome was then annotated using the standalone Prokaryotic Genome Annotation Pipeline (PGAP) version 2019-08-01.build3919 (11). A total of 4,422 genes were predicted, including 4,280 coding sequences (CDS) and 142 RNA genes. Among the RNA genes, 25 were rRNA genes (9 5S rRNA, 8 16S rRNA, and 8 23S rRNA), and 112 were tRNA genes. Protein sequences were annotated using the BlastKOALA (12) tool (accessed January 2019) for Kyoto Encyclopedia of Genes and Genomes (KEGG) enzyme codes. The proteins were also clustered on the basis of homology using the annotation resource Clusters of Orthologous Groups (COG) database (accessed January 2019) (13). In total, 2,219 and 2,910 genes were assigned to KEGG orthology (KO) and COG categories, respectively. About 1,275 protein-coding genes were connected to KEGG pathways, and 1,172 genes encoded enzymes mapping to Enzyme Classification numbers. Default parameters were used to run all the software/tools unless otherwise specified.

Future investigations may delineate the role of *B. marisflavi* strain P3 as a potential biofertilizer, similar to other members of the *Bacillus* genus.

### Data availability.

This whole-genome shotgun project has been deposited at DDBJ/EMBL/GenBank under the accession number VSJG00000000. The version described in this paper is version VSJG00000000.1. The BioProject accession number is PRJNA478293.
